# Delay in diagnosis of muscle disorders depends on the subspecialty of the initially consulted physician

**DOI:** 10.1186/1472-6963-11-91

**Published:** 2011-05-04

**Authors:** Simone Spuler, Andrea Stroux, Franziska Kuschel, Adelheid Kuhlmey, Friederike Kendel

**Affiliations:** 1 Muscle Research Group, Charité - Universitätsmedizin Berlin, Berlin, Germany; 2 Department of Biometry and Clinical Epidemiology, Charité - Universitätsmedizin Berlin, Berlin, Germany; 3 Institute of Medical Sociology, Charité - Universitätsmedizin Berlin, Berlin, Germany; 4 Institute of Medical Psychology, Charité - Universitätsmedizin Berlin, Berlin, Germany

## Abstract

**Background:**

New therapeutic strategies in muscular dystrophies will make a difference in prognosis only if they are begun early in the course of the disease. Therefore, we investigated factors that influence the time to diagnosis in muscle dystrophy patients.

**Methods:**

A sample of 101 patients (mean age 49 years; range 19-80; 44% women) with diagnosed muscle dystrophies from neurological practices and the neuromuscular specialty clinic in Berlin, Germany, was invited to participate. Time from first consultation to diagnosis, subspecialty of physician, and sociodemographic data were assessed with self-report questionnaires. The association between time to diagnosis and potential predictors (subspecialty of initially consulted physician, diagnoses, gender, and age at onset) was modeled with linear regression analysis.

**Results:**

The mean time span between first health-care contact and diagnosis was 4.3 years (median 1). The diagnostic delay was significantly longer if patients were initially seen by a non-neurological specialist compared to a general practitioner (5.2 vs. 3.5 years, p = 0.047). Other factors that were independently associated with diagnostic delay were female gender and inherited muscle disease.

**Conclusion:**

Action to improve clinical awareness of muscle diseases in non-neurological specialists is needed.

## Background

Our mechanistic understanding of muscular dystrophies and inherited myopathies has improved remarkably during the last decade [1]. This progress leads to a much higher level of diagnostic accuracy and to the possibility of new therapeutic strategies [2,3]. To benefit from this progress, the diagnosis of a muscle disorder must be made early. However, certain factors may contribute to delay in diagnosis. First, the term "neuromuscular disorder" encompasses more than 300 different rare chronic diseases. Second, myopathies and muscular dystrophies present with unspecific complaints, such as difficulty climbing stairs, heaviness in the legs, myalgias, or cramps. The time course is often slow and years may pass before a significant worsening is perceived. Many different subspecialties from primary care, internal medicine, orthopedics, rheumatology, or psychosomatic medicine may be consulted and may give various tentative diagnoses before the patient is referred to a neurologist. For example, fatigue may be mistakenly interpreted as lack of exercise or diminished motivation. An elevated level of creatine kinase level, when determined at all, can be misinterpreted as heart disease. Some health care systems, like the British, allow access to medical specialists only after consultation of a general practitioner (GP). In other medical systems, such as the one in Germany, the choice is left to the patient whether a GP or a specialist serves as the primary contact. In any event, a neurologist is seldom the first choice.

The medical and economic consequences of these different systems are poorly studied. The time from the first consultation to the correct diagnosis in patients with amyotrophic lateral sclerosis (ALS) [4,5] has been investigated, as has the course of Duchenne muscle dystrophy (DMD) patients [6]. Other studies have examined the time from first onset to diagnosis in patients with myasthenia gravis [7], body inclusion myositis (IBM) [8], and Becker's congenital myotonia [9]. The experience in ALS patients suggests that gender and subspecialty of medical doctors may contribute to the diagnostic delay [4]. However, there are few data on the broad muscular dystrophy spectrum in adult patients. We therefore investigated factors that influence the time to diagnosis in muscle dystrophy patients.

## Methods

### Study setting and sample

Patients were recruited into the study from neurologists in private practice, the patient support group of the German Muscle Society, or from the neuromuscular specialty clinic at the Charité University Hospital, Berlin. Inclusion criteria were a diagnosis of muscle dystrophy, and age above 18 years. There were no patients with DMD. All patients enlisted voluntarily in the study and provided informed consent after due Institutional Review Board approval by the Charité - Universitätsmedizin Berlin.

### Materials and procedure

We gave the patients standardized self-report questionnaires. We asked them about their diagnosis, the initial choice of medical practitioner, the time interval between first medical consultation and the definitive diagnosis, and the nature and number of referrals. Questionnaires were anonymized and approved by the data security engineer. The response rate was 104 out of 130 (80%). Three patients were excluded from the analysis because they failed to report on the time of the first medical consultation and time of diagnosis. Thus, our final sample comprised 101 adult patients (44 women). The medical subspecialties were divided into three categories: (1) general practitioners (GP), (2) non-neurological specialists (orthopedic surgeons, internists, physical medicine and rehabilitation specialists, psychosomatic medicine, and rheumatologists), and (3) neurologists.

### Data analysis

Comparisons among men and women were made using the Mann-Whitney U test or the Student's t-test for continuous variables depending on the distribution of data, and the *χ^2^*-square test for categorical variables. Mann-Whitney U test (two groups) or Kruskal-Wallis test (more than two groups) were used to determine differences in time to diagnosis among different subspecialties. Multiple linear regression analysis with forward selection was performed to assess associations between time to diagnosis and a set of predictors. A square root transformation was fitted to the variable "time to diagnosis" to approximate the data to a normal distribution. A two-tailed alpha level of 0.05 was considered significant for all analyses. All analyses were performed using SPSS 16.0 (SPSS Inc.; Chicago, IL).

## Results

The average age of the 101 study participants was 48.9 years (SD = 14.5) with a range of 19 to 80 years. The sample was relatively well educated with over 90% of participants reporting a medium or high level of education. More than half of the patients had an inherited muscle disease, and in 36% of the patients, the onset of disease occurred in childhood or adolescence (Table 1).

**Table 1:** Sample characteristics

		Entire sample (N = 101)	Men (n = 57)	Women (n = 44)	p
Age (yrs); mean ± SD		48.9 ± 14.5	49.2 ± 14.6	48.6 ± 14.8	0.854

Education level	Low	7 (7.1%)	3 (5.4%)	4 (9.3%)	0.614
	Medium	46 (46.5%)	28 (50.0%)	18 (41.9%)	
	High	46 (46.5%)	25 (44.6%)	21 (48.8%)	

Age group at onset	Childhood /adolescence	36 (35.6%)	19 (33.3%)	17 (38.6%)	0.581
	Adulthood	65 (64.4%)	66.7 (67%)	27 (61.4%)	

Diagnoses	Inherited muscle diseases*	56 (55.4%)	31 (54.4%)	25 (56.8%)	0.807
	Acquired muscle diseases**	22 (21.8%)	10 (17.5%)	12 (27.3%)	0.240
	SMA III and IV***	14 (13.9%)	8 (14%)	6 (13.6%)	0.954

Time from first consultation to diagnosis (yrs); mean (median)		4.3 (1.0)	2.9 (1.0)	6.1 (3.0)	0.014

First consultation	Neurologists	15 (14.9%)	9 (15.8%)	6 (13.6%)	0.763
	General practitioners	38 (37.6%)	24 (42.1%)	14 (31.8%)	0.29
	Non-neurological specialists	48 (47.5%)	24 (42.1%)	24 (54.5%)	0.214

Note. SD = standard deviation

*limb-girdle muscular dystrophies (MD), facioscapulohumeral MD, AD Emery Dreifuss MD, congenital myopathies, metabolic myopathies, channelopathies, no patients with Duchenne MD are included

**inclusion body myositis, polymyositis

***spinal muscle atrophy

### The primary health care step and time to diagnosis

**Figure 1:**
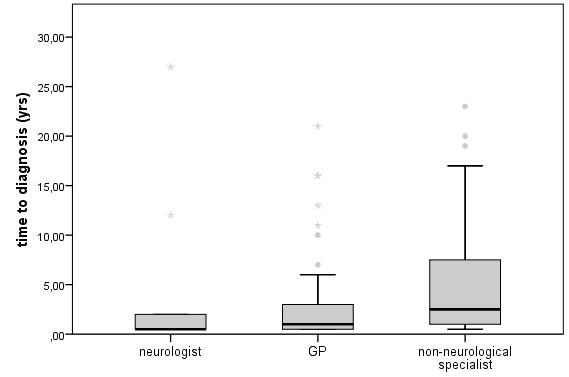
**Displayed are differences in diagnostic delay according to medical subspecialty at first consultation**. The delay is longest for patients who first consulted a non-neurological specialist (e.g. internist, orthopedics). Boxes display values between 25 and 75 percentile; outliers are indicated by stars.

Thirty eight patients first consulted a GP after the onset of myopathy-related symptoms, whereas 48 patients chose non-neurological specialists. Only 15 patients consulted a neurologist as the primary health care step (Table 1). These patients either had family members with a hereditary muscle disorder or had an above-average level of education (some were physicians themselves). Even in this group, only 60% of the patients were diagnosed within one year. The mean time between first consultation of a physician and the definite diagnosis was 4.3 years (MD = 1) (Table 1). The time to diagnosis (Figure 1) was dependent on subspecialty of physician with a significant difference between the three groups (p = 0.021). When a neurologist or a GP was initially consulted, the time from first visit to diagnosis was 3.4 years (MD = 0.5) and 3.5 years (MD = 1), respectively. When a non-neurological specialist was the first medical contact, the time to diagnosis increased to 5.2 years (MD = 2.5). Subgroup analyses showed a significant difference in diagnostic delay between a GP und non-neurological specialists in that the GPs were better (p = 0.047) and between neurologists and non-neurological specialists (p = 0.016). After the initial consultation, 18 patients were misdiagnosed and treated for prolonged periods for some other presumed condition. Non-neurologists initially managed 17 of these 18 patients. GPs immediately referred 17 (45%) of their patients to a neuromuscular center, and 20 (53%) to a non-neurological specialist.

### Gender differences in diagnostic delay

**Figure 2:**
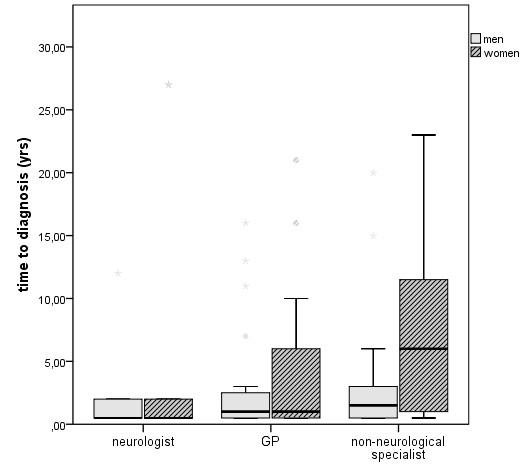
**Association between medical subspecialty at first health care action, gender, and diagnostic delay is shown**. Time span between first medical consultation and diagnosis is increased for patients initially seen by a non-neurological specialist. The delay is longest for women who first consulted a non-neurological specialist. Boxes display values between 25 and 75 percentile; outliers are indicated by stars.

Forty-four percent of subjects were women. There were no gender differences in education, age at onset, diagnoses, or specialty of first consultation (Table 1). However, for women the time from first consultation resulting from myopathy-related symptoms to diagnosis was significantly longer compared to men (p = 0.014) (Table 1). This effect was particularly striking when non-neurological specialists were consulted in the first instance (7.2 years for women vs. 3.2 years for men, p = 0.015) (Figure 2).

### Predictors for time to diagnosis

Results from univariable regression analyses showed that age at onset, inherited muscle disease, female gender, and subspecialty were significantly associated with time to diagnosis (Table 2). Multiple regression analysis with forward selection, including type of muscle disease, education, gender, subspecialty of first consultation, and age at onset, revealed that only inherited muscle disease, female gender, and subspecialty were independent predictors of time to diagnosis (Table 2).

**Table 2:** Associations with time to diagnosis: results from univariable and multiple regression analysis.

Variable	Univariable regression			Multiple regression*		
	β**	**95% CI**	**p-value**	β**	**95% CI**	**p-value**

Gender	0.276	0.202 - 1.127	0.005	0.281	0.212 - 1.152	0.008
Education	0.115	-0.166 - 0.616	0.257			
Age at onset	0.234	0.101 - 1.069	0.018			
**Type of disease**						
Inherited muscle diseases	0.212	0.041 - 0.979	0.033	0.260	0.170 - 1.088	0.008
Acquired muscle diseases	-0.165	-1.048 - 0.092	0.099			
SMA III and IV	-0.099	-1.031 - 0.343	0.323			
**Subspecialty**						
Neurologist	-0.120	-1.071 - 0.261	0.230			
General practitioner	-0.113	-0.769 - 0.209	0.259			
Non-neurological specialist	0.196	0.01 - 0.937	0.05	0.226	0.085 - 1.004	0.021

Note. β = standardized regression coefficient; CI = confidence interval; SMA = spinal muscle atrophy

*multiple linear regression analysis with forward selection

## Discussion

Our study found two key results. First, patients consulting a non-neurological specialist experience a longer delay in diagnosis, compared to patients who initially consulted a neurologist or a GP. Second, for women, the diagnostic delay was even longer than for men. Our data showed that there was no difference in time to diagnosis when initially either a neurologist or a GP was consulted. Interestingly, first consulting another subspecialty resulted in a significant diagnostic delay compared to a neurologist or a GP. In Germany, the choice is left to the patient as to whether a GP or a specialist serves as the primary contact. Other health care systems allow access to a specialist only after consultation with a GP. Leaving the patient with the decision to choose a medical subspecialist as the primary point of contact with the medical profession has profound effects on the diagnostic course. Our data support the usefulness of a system that first requires the consultation of a GP, followed by assignment to a specialist. The GP appeared to be well qualified by training to direct the patient to the correct subspecialty.

Our results are consistent with findings indicating that patients with predominantly adult-onset muscle diseases experience a diagnostic delay that is several years longer than the life-span observed in boys with DMD (mean delay 1 year 11 months) [10,11]. One reason may be the more benign and subtle course of many muscular dystrophies and myopathies, which may be associated with less-obvious symptoms than dystrophinopathies. Motor neuron diseases such as ALS progress more rapidly and are correctly diagnosed within a time span of 11 and 13 months, as indicated by other studies [4,5]. Another reason may be related to the lower prevalence of limb girdle muscular dystrophies and other myopathies, compared to DMD [12]. This state of affairs could lead to a very low level of clinical awareness. Generally, rare diseases are less thoroughly emphasized in medical school. Moreover, the infrequent contact with muscle diseases in clinical praxis may not sufficiently consolidate these cases in the physician's memory. Inherited diseases showed a longer time from first contact to definite diagnosis, compared to acquired diseases. Often, inherited muscle diseases present with a subtle course, whereas symptoms accelerate more rapidly in acquired diseases. Although one would expect a higher awareness in patients with affected relatives, patients often are not aware that family members have the disease [13]. Alternatively, the disease may be less prevalent in the family due to being passed on a recessive chromosome.

The diagnostic delay affected women more than men, particularly when the women were initially seen by non-neurological specialists. Several factors may contribute to this effect. First, unspecific symptoms like heaviness of the legs, tiredness, or cramps may not be taken as seriously in women compared to men. We know from other studies that symptoms such as fatigability are more often attributed to psychological problems in women than in men [14,15]. Also, men are physically more active and therefore might be more quickly aware of physical problems. In addition, men have a tendency to wait longer until consulting a physician and are thus more likely to present with a more advanced disease [16], which then may be easier to diagnose.

We are aware of limitations. First, patients might differ in their ability to recall information about their first consultation and the correct time point of the diagnosis. Second, due to the rareness of muscular dystrophies, our sample is comparatively small. Third, the waiting time for an appointment was not assessed in this investigation. However, since waiting time for an appointment is usually less than three months in Germany, this issue seems negligible.

## Conclusions

Any future therapeutic strategy will probably only make a real difference in prognosis if the treatment is begun early in the course of the disease [17]. Whereas in DMD patients pediatricians are primarily involved in terms of newborn-screening and other related programs [18], adult-onset muscle diseases can present to a variety of medical subspecialties. Neurologists and neuromuscular specialists should make an active effort to increase the clinical awareness of muscle diseases among other medical specialties.

## Competing interests

The authors declare that they have no competing interests.

## Authors' contributions

SSpu originated the idea for this study, designed the study, enrolled the patients, analysed the data, and prepared the manuscript. AS participated in the data analysis and in the interpretation of data. FKu delineated the standardized questionnaire, contributed to the enrollment of patients, and participated in the interpretation of data. AK participated in the interpretation of the data and in the discussion of the paper. FK participated in the data analyses, in the interpretation of data and the preparation of the manuscript. All authors read and approved the final manuscript.

## Pre-publication history

The pre-publication history for this paper can be accessed here:

http://www.biomedcentral.com/1472-6963/11/91/prepub
